# Interventions for developing selective and sustained attention in adolescent athletes: a scoping review

**DOI:** 10.3389/fpsyg.2026.1816887

**Published:** 2026-09-21

**Authors:** Solvita Pleinica, Sanita Šuriņa, Guna Semjonova, Gints Peleckis, Jason Tang, Agris Liepa

**Affiliations:** 1 Department of Psychology, Rīga Stradiņš University, Rīga, Latvia; 2 Department of Rehabilitation, Rīga Stradiņš University, Rīga, Latvia; 3 Department of Individual and Team Sports, Rīga Stradiņš University, Rīga, Latvia; 4 School of Management, University of Bath, Bath, United Kingdom

**Keywords:** adolescent, athletes, selective attention, sport, sustained attention

## Abstract

**Introduction:**

Selective and sustained attention are key cognitive functions associated with athletic performance and represent trainable abilities during adolescence. Although various intervention approaches have been investigated to enhance attention in young athletes, the available evidence has not been comprehensively mapped. Therefore, the aim of this scoping review was to identify and map the available interventions designed to develop selective and sustained attention among adolescent athletes and to summarize their characteristics, assessment methods, and reported outcomes.

**Methods:**

The review followed the Preferred Reporting Items for Systematic Reviews and Meta-Analyses Extension for Scoping Reviews (PRISMA-ScR) guidelines. The research question was developed using the Population–Concept–Context (PCC) framework. A systematic search of six electronic databases (ScienceDirect, ProQuest, PubMed, Scopus, Cochrane Library, and EBSCOhost) was conducted in December 2025. Data were extracted using Microsoft Excel and synthesized narratively.

**Results:**

Six studies met the inclusion criteria, comprising 393 participants. Among the 367 participants for whom sex/gender was reported, 170 (46.3%) were female. The included studies primarily focused on adolescent athletes aged 11–20 years; one study included a broader age range, although its intervention group corresponded to the target adolescent population. The identified interventions included cognitive training, cognitive-motor dual-task training, mindfulness-based interventions, perceptual-cognitive training, and sport-specific approaches targeting selective and/or sustained attention. A variety of assessment instruments and measurement methods were used to evaluate attention-related and other cognitive outcomes. Most studies reported improvements in selective and/or sustained attention following the interventions; however, substantial heterogeneity in intervention characteristics, outcome measures, and study designs limited direct comparisons across studies.

**Discussion:**

The available evidence indicates that several intervention approaches have been explored for developing selective and sustained attention in adolescent athletes. However, the small number of studies and considerable methodological heterogeneity preclude firm conclusions regarding intervention effectiveness. Future research should prioritize well-designed intervention studies with larger samples, standardized intervention protocols, and more consistent attention assessment methods.

## 1 Introduction

Early sport specialization has become increasingly common, accompanied by growing recognition of the importance of cognitive factors for athletic performance (Wilczyńska et al., 2022). Among these factors, attention plays a central role because athletes are continuously required to detect relevant information, ignore distractions, and make rapid decisions under changing environmental conditions (Voss et al., 2010). Consequently, insufficient development of attentional abilities may impair perceptual processing, decision-making, and overall sport performance.

Attention is a fundamental cognitive process that enables individuals to selectively allocate mental resources to relevant environmental stimuli while suppressing competing information (Styles, 2006; Kondrova, 2010). Among its different components, selective attention and sustained attention are particularly important in sport. Selective attention enables athletes to prioritize relevant cues while filtering irrelevant information, thereby supporting anticipation and decision-making (Abernethy, 2001; Memmert, 2009; Moran, 2005; Chun et al., 2011; Müller and Krummenacher, 2006). Sustained attention refers to the ability to maintain attentional focus over prolonged periods despite fatigue or monotonous demands and is essential for maintaining consistent performance throughout training and competition (Esterman and Rothlein, 2019; Unsworth and Robison, 2020; Luque-Casado et al., 2016; Esterman et al., 2013, 2014). Although conceptually distinct, these attentional processes operate simultaneously during sport performance and should therefore be considered together.

Adolescence represents a particularly important period for attention development because executive functions and attentional control continue to mature throughout this developmental stage (Betts et al., 2006; Lewis et al., 2017; McAvinue et al., 2012). At the same time, competitive sport places progressively greater cognitive demands on young athletes, requiring rapid information processing, anticipation, and decision-making. Previous research suggests that even relatively short cognitive interventions may improve attentional performance during adolescence (Visocka, 2002), highlighting the potential value of systematic attention training within organized sport.

Several intervention approaches have been investigated to improve attentional performance in athletes. Cognitive training programmes, Cognitive-Motor Dual-Task (CMDT) training, and mindfulness-based interventions are described together with their theoretical rationale and relevance for sport performance. Although these intervention approaches differ considerably in their theoretical foundations and implementation, they all seek to enhance attentional performance relevant to sport.

Despite the growing variety of intervention approaches, the available evidence remains fragmented. The included studies demonstrated substantial heterogeneity in participant characteristics, including age, sport type, intervention duration, training frequency, theoretical framework, and attention assessment methods, making direct comparison difficult. Furthermore, selective attention has been investigated considerably more frequently than sustained attention, despite both constructs being essential for successful sport performance. Interpretation of intervention effects is further complicated by the fact that selective and sustained attention are dynamic cognitive functions that continue to develop throughout adolescence and are influenced by ongoing neurocognitive maturation, making intervention studies in this population particularly challenging (Betts et al., 2006; Lewis et al., 2017; McAvinue et al., 2012). Considerable variability also exists in the cognitive instruments used to evaluate intervention effectiveness, limiting comparisons across studies and making it difficult to identify the most appropriate assessment methods (Fleddermann et al., 2019; Lucia et al., 2022).

To date, no scoping review has systematically synthesized interventions specifically targeting selective and sustained attention in adolescent athletes. Consequently, there is currently no comprehensive overview of which intervention approaches have been investigated, which attention assessment instruments have been used, and how intervention outcomes have been reported across studies. Such evidence is needed to identify current knowledge gaps, support future intervention development, and inform researchers, sport psychologists, coaches, and youth sport practitioners.

Therefore, the aim of this scoping review was to identify and map the available interventions designed to develop selective and sustained attention among adolescent athletes and to summarize their characteristics, assessment methods, and reported outcomes. Specifically, this review aimed to (1) identify and categorize the intervention approaches used to develop selective and sustained attention, (2) summarize the assessment instruments used to measure selective and sustained attention, and (3) describe the reported outcomes of these interventions. Accordingly, the primary research question was: *What interventions have been used to develop selective and sustained attention among adolescent athletes?* Secondary research questions were: (1) *What assessment instruments have been used to measure selective and sustained attention?* and (2) *What outcomes have been reported following these interventions?*

## 2 Methods

This scoping review was conducted following the methodological framework proposed by Arksey and O’Malley (2005), incorporating the methodological refinements proposed by Levac et al. (2010) and the guidance provided in the JBI Manual for Evidence Synthesis (Peters et al., 2020). The review was reported in accordance with the Preferred Reporting Items for Systematic Reviews and Meta-Analyses extension for Scoping Reviews (PRISMA-ScR) guidelines (Tricco et al., 2018).

Step 1: Identifying the research questions. An initial exploratory search of the literature was conducted to determine the scope of existing research on interventions targeting selective and sustained attention in adolescent athletes. The research questions were subsequently formulated using the Population–Concept–Context (PCC) framework recommended for scoping reviews (Peters et al., 2020), where the Population comprised adolescent athletes, the Concept referred to interventions targeting selective and sustained attention, and the Context was organized sport settings. Because this review followed a scoping review methodology, the Population–Concept–Context (PCC) framework was used instead of the Population, Intervention, Comparison, Outcome, and Study Design (PICOS) framework, in accordance with the recommendations of the Joanna Briggs Institute (Peters et al., 2020). The PCC framework guided the development of the research questions, eligibility criteria, search strategy, and data-charting process.

Step 2: Identifying relevant studies – Search strategy. A systematic literature search was conducted in December 2025 using six electronic databases: *PubMed, Scopus, ScienceDirect, ProQuest, EBSCOhost, and the Cochrane Library.*

The search strategy was developed before the literature search and was based on keywords related to selective attention, sustained attention, vigilance, and sport. An initial search strategy was developed for PubMed and subsequently adapted for each database according to its indexing system, controlled vocabulary, search syntax, Boolean operators, truncation symbols, and field tags while maintaining conceptual equivalence across all database searches.

Preliminary searches including intervention-related terms (e.g., intervention, training, and attention training) were conducted; however, these substantially reduced the retrieval of relevant studies. Therefore, a broader search strategy was adopted to maximize search sensitivity, while intervention-specific eligibility was ensured during the study selection process through the predefined inclusion criteria and full-text screening.

The following search strategy formed the basis of all database searches: *(“selective attention” OR “sustained attention” OR vigilance OR attention) AND (athlete OR sport) AND (adolescents OR youth) NOT (“attention deficit hyperactivity disorder” OR ADHD OR anxiety OR depression).* The search syntax was adapted to the requirements of each database, and the complete search strategies are presented in Table 1.

**TABLE 1:** Database-specific search strategies used in the scoping review.

Main database	Database	Keywords	Search within
*ProQuest*		(“sustained attention” or “selective attention” or “vigilance” or attention) AND (“athletes” or “athletes” or “athlete” or “athlete” or sport) AND (adolescents or youth) NOT (anxiety or depression or ADHD or Attention Deficit Hyperactivity Disorder)	All subjects and indexing – subject
*PubMed*	MEDLINE	(“sustained attention” or “selective attention” or “vigilance” or attention) AND (“athlete s” or “athletes” or “athlete” or “athlete” or sport) AND (adolescents or youth) NOT (anxiety or depression or ADHD or Attention Deficit Hyperactivity Disorder)	–
*EBSCO*	MEDLINE ultimate; sociology source ultimate; health source- consumer edition; health source: nursing/academic edition; MEDLINE	(“sustained attention” or “selective attention” or “vigilance” or attention) AND (“athlete s” or “athletes” or “athlete” or “athlete” or sport) AND (adolescents or youth) NOT (anxiety or depression or ADHD or Attention Deficit Hyperactivity Disorder)	Su subject terms
*Science Direct*	–	(“sustained attention” or “selective attention” or “vigilance” or attention) AND (“athlete s” or “athletes” or “athlete” or “athlete” or sport) AND (adolescents or youth) NOT (anxiety or depression or ADHD or Attention Deficit Hyperactivity Disorder)	–
*Scopus*	–	(“sustained attention” or “selective attention” or “vigilance” or attention) AND (“athlete s” or “athletes” or “athlete” or “athlete” or sport) AND (adolescents or youth) NOT (anxiety or depression or ADHD or Attention Deficit Hyperactivity Disorder)	Article title, abstract, keywords
*Cochran*	Ct.gov; CINAHL	(“sustained attention” or “selective attention” or “vigilance” or attention) AND (“athlete s” or “athletes” or “athlete” or “athlete” or sport) AND (adolescents or youth) NOT (anxiety or depression or ADHD or Attention Deficit Hyperactivity Disorder)	Title, abstract, keyword

Step 3: Study selection. Eligibility criteria were established before the study selection process using the PCC framework.

Studies were included if they met all of the following criteria: (1) Participants were adolescent athletes aged 11–20 years participating in organized sport; (2) The study evaluated an intervention specifically designed to develop selective attention, sustained attention, or both; (3) The intervention lasted longer than 1 week and included at least four intervention sessions; (4) Selective and/or sustained attention was assessed before and after the intervention, during the intervention, and/or through comparison with a control group using objective and/or subjective outcome measures; (5) The study employed a quantitative research design; (6) The article was published in English; (7) The study was published between 1 January 2000 and 1 December 2025.

Only studies for which the full text was accessible through institutional subscriptions or open-access sources were assessed for eligibility. Studies for which the full text could not be accessed were excluded because eligibility and data extraction could not be completed.

Studies were excluded if they: (1) investigated participants with attention deficit hyperactivity disorder (ADHD), depression, anxiety, or other neurological, psychiatric, or health-related conditions; (2) evaluated attentional bias modification or attention-training interventions designed primarily for clinical or psychological treatment; (3) were systematic reviews, scoping reviews, review articles, study protocols, conference abstracts, editorials, or secondary analyses; or (4) did not provide sufficient methodological information for data extraction. The review focused on generally healthy adolescent athletes participating in organized sport. Therefore, studies involving clinical populations were excluded, as interventions targeting individuals with ADHD, anxiety, depression, or other neurological or psychiatric conditions are typically designed for therapeutic purposes rather than for enhancing selective or sustained attention in healthy athletes.

All records identified through the database searches were exported to the systematic review platform *Rayyan* (Ouzzani et al., 2016), where duplicate records were identified and removed. Study selection was conducted in two stages. First, titles and abstracts were screened against the predefined eligibility criteria. Subsequently, the full texts of potentially eligible studies were assessed for inclusion. The study selection process, data charting, and data synthesis were conducted in accordance with the predefined review protocol developed before the literature search commenced. The study selection process followed the PRISMA-ScR guidelines and is presented in the PRISMA-ScR flow diagram (Figure 1).

**FIGURE 1:**
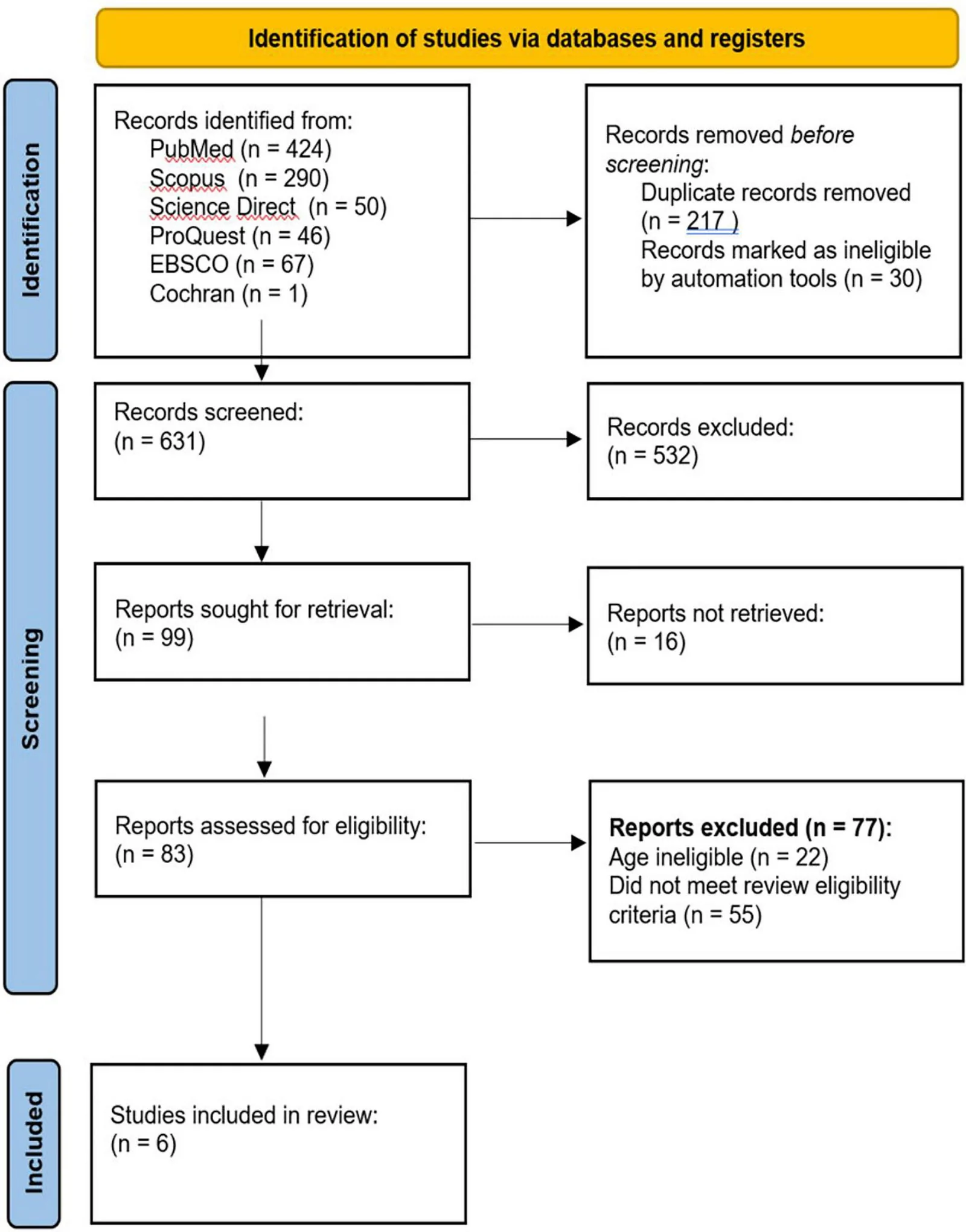
PRISMA 2020 flow diagram of the study identification and selection process, adapted from Page et al. (2021) under the CC BY 4.0 license.

Step 4: Charting the data. The selected studies were documented using Excel tables. First, data were collected from the studies and grouped into tables. In the next step, the extracted data were compared and cross-checked against the information reported in the included studies. Key characteristics of the included studies were identified, such as author(s); year of publication; study title; study aim; country; sport; study design; theory and type of attention (see Table 2). Table 3 shows an overview of all selected studies, with information on first author, year of publication; population; age; type of attention; experimental group intervention; control group intervention; primary and secondary measures. Table 4 summarizes the data showing first author; type of attention; intervention period; total session time, including total minutes and intervention components.

**TABLE 2:** Key characteristics of the included studies for intervention to develop selective and sustained attention.

References	Theme	Aim	Country	Type of sport	Design	Theoretical framework (if reported)	Type of attention
Dania et al., 2021	Chess training for improving executive functions and invasion game tactical behavior of college student athletes: a preliminary investigation.	The aim of this study was to explore whether a chess training could affect intervention game athletes EFs and tactical game performance.	Greece	Students of Physical Education and Sport Science with a background in team sports basketball, soccer and handball.	A pretest-posttest quasi-experimental design with a control group.	Anderson’s active control of thought model. Executive functions.	SelA (including RI, WM, CF components)
Fleddermann et al., 2019	Off-court generic perceptual-cognitive training in elite volleyball athletes: task-specific effects and levels of transfer.	The aim of this study was to examine the efficacy of a generic off-court perceptual-cognitive training in elite volleyball players on three different levels: task-specific, near-transfer, and far-transfer effects.	Germany	Volleyball.	An interventional design.	Perceptual-cognitive expertise.	SusA (MS, WS, PS).
Kittler et al., 2022	Effects of a mindfulness-based intervention on sustained and selective attention in young top-level athletes in a school training setting: a randomized control trial study	To evaluate the effectiveness of a mindfulness - based intervention on attention in young athletes.	Germany	Archery, basketball, boxing, fencing, figure skating, football, gymnastics, soccer, ice dancing, hockey, judo, speed skating, swimming, athletics, volleyball, weightlifting.	A randomized controlled trial (waitlist -controlled crossover design).	Mindfulness - based practice models.	SelA, SusA.
Lola et al., 2023	The influence of analogies on the development of selective attention in novices in normal or stressful conditions.	The effect of explicit, implicit, and analogy learning methods on developing selective attention skills in young novice participants was examined under normal and stressful conditions.	Greece	Volleyball	A randomized controlled trial.	Attentional control theory.	SelA.
Lucia et al., 2022	Specific effect of a cognitive-motor dual-task training on sport performance and brain processing associated with decision-making in semi-elite basketball players.	Aimed at evaluating the effects of cognitive-motor dual-task training (CMDT) on sport-specific athletic performance and cognitive functions of semi-elite basketball players.	Italy	Basketball	A randomized controlled trial.	Dual-task model.	SelA.
Reigal et al., 2019	Effects of a computerized training on attentional capacity of young soccer players.	To assess the effects of a 9 week computerized program on selective attention in a group of young soccer players.	Spain	Soccer	A quasi-experimental design.	Not specified	SelA.

SelA, selective attention; SusA, sustained attention; RI, response inhibition; WM, working memory; CF, cognitive flexibility; MS, memory span; WS, working speed; PS, processing speed.

**TABLE 3:** Overview of all selected studies for intervention to develop selective and sustained attention.

References	Population	Age	Type of attention	IG intervention	Control group intervention	Assessment instruments and measurement methods
Dania et al., 2021	Students of physical education and sport science *N* = 26, IG (*n* = 13), CG (*n* = 13).	19–20 *M* = 20.58, SD = 1.74	SelA (RI, WM, CF).	Executive function - chess training.	Received no specific treatment.	Executive functions: modified Eriksen Flanker Task (selective attention/interference control; PsyToolkit); working memory and cognitive flexibility tasks (PsyToolkit); tactical game performance: game performance assessment instrument (GPAI)
Fleddermann et al., 2019	(Inter)national (beach-) volleyball athletes *N* = 43, IG (*N* = 22, *n* = 2 males, *n* = 20 females), CG (*N* = 21, *n* = 5 males, *n* = 16 females).	14–26 (adolescent subgroup included) IG *M* = 16.38, SD = 1.7, CG *M* = 21.38, SD = 4.53	SusA (MS, WS, PS).	Completed–in addition to regular ball and athletic practice–a specific dynamic PCT with the 3D-MOT.	Progressed with their regular practice, such as ball and athletic practice.	Task-specific practice test: 3D-Mot task with the NeuroTracker Core Program by CogniSens Athletics Inc. from the University of Monreal; Near-transfer tasks (cognitive tests): (a) SuSA (d2-R), (b) MS (KAI-N), (c) WS (KAI-N), (d) PS Zahlenverbindungstest (ZVT); Far- transfer tasks (volleyball- specific test) - block jumps tasks: (a) single- task, (b) Dual- task low;(c) Dual-task high.
Kittler et al., 2022	Sport school students *N* = 137, *n* = 46 female, *n* = 91 male. IG (*n* = 68), CG (*n* = 69).	12–13 *M* = 12,2, SD = 0,50	SelA and SusA.	BATL, regular physical education classes.	BATL mindfulness program. Regular physical education classes.	Frankfurter Aufmerksamkeits-Inventar 2 (FAIR-2), a Go/No-Go task assessing selective attention through quantity (L) and quality (Q) scores and sustained attention through the continuity/performance score (K).
Lola et al., 2023	Volleyball novice players *N* = 60 female, (a) ExG (*n* = 15), (b) ImG (*n* = 15), (c) AnG (*n* = 15), (d) CG (*n* = 15).	11–12	SelA.	The video simulation intervention program.	Only participated in the measurements.	Selective-attention simulation task using volleyball-specific visual stimuli; reaction time and response accuracy recorded with SuperLab software; modified Competitive State Anxiety Inventory-2 (CSAI-2) for cognitive and somatic anxiety/stress.
Lucia et al., 2022	Semi-elite basketball players *N* = 52; *n* = 28 females; *n* = 24 males. EG (*n* = 26); CG (*n* = 26).	15–18 *n* = 28 females (*M* = 16.1, SD = 1.1 years); *n* = 24 males (*M* = 16.5, SD = 1.2 years)	SelA.	Basketball match, basketball team training, individual CMDT.	Basketball match, basketball team training, individual motor training.	Sport-specific performance: basketball test; Cognitive performance: visuomotor discrimination task (DRT/Go-No-Go task), including response accuracy and median response time (RT); neurophysiological assessment: EEG recording using BrainVision; eye movements monitored by electrooculography (EOG).
Reigal et al., 2019	Two soccer club players *N* = 75 male, EG (*n* = 37), CG (*n* = 38).	14–18 *M* = 15.45; SD = 1.43	SelA.	Attention training program - Rejilla v. 1.0 software, performed eight tasks.	Did not participate in the attention training program and did not perform alternative tasks, although they did continue training soccer.	D2 attention test; Rejilla 1.0 software (eight tasks).

SelA, selective attention; SusA, sustained attention; IG, intervention group; EG, experimental group; CG, control group; ExG, explicit learning group; ImG, implicit learning group; AnG, analogy learning group; PCT, perceptual-cognitive training; 3D-MOT, 3D - Multiple Object Tracking task; BATL, Berliner Achtsamkeitstraining zur Leistungsoptimierung, Berlin Mindfulness Based Training for Athletes; CMDT, cognitive-motor dual-tasking training; RI, response inhibition; WM, working memory; CF, cognitive flexibility; MS, memory span; WS, working speed; PS, processing speed.

**TABLE 4:** Intervention specification and components of the selected studies.

References	Type of attention	Intervention period	Total sessions	Intervention
Dania et al., 2021	SelA (RI, WM, CF).	10 weeks, one unit per week, each lasting 2 h.	10 sessions 20 h	Executive function: chess game with thematic units: introduction to chess thinking; strategic moves and concepts; time control and management; decision making; time regulation; problem solving I; problem solving II; creativity.
Fleddermann et al., 2019	SusA (MS, WS, PS).	8 weeks, two units per week, each lasting 30 min.	16 units 8 h	Completed–in addition to regular ball and athletic practice–a specific dynamic PCT with the 3D-MOT.
Kittler et al., 2022	SelA, SusA.	6-week mindfulness programme, one session per week (60 min); long-term follow-up approximately 33 weeks after the intervention.	Six BATL sessions; 360 min (6 h) in total	BATL mindfulness program. Regular physical education classes.
Lola et al., 2023	SelA.	4 weeks, three units per week, each lasting 30 min.	12 sessions, 6 h.	The video simulation intervention program: ExG - watched edited video clips from official volleyball matches, which showed the player performing the serve and the reception of the opposing team. In every video clip, the coach gave one (1) instruction out of ten (10). ImG - watched the same videos of expert players executing volleyball serves as the ExG, but they also had to respond to a secondary dual cognitive task through random letter generation. AnG - watched the same video clips of expert players executing volleyball serves. The coach gave the same number (10) of instructions using a metaphor.
Lucia et al., 2022	SelA	EG - 5 weeks, seven units per week, 1 day for a basketball match (2 h) and six times a week for standard basketball training with group basketball training (3 h) and two standard individual training sessions (30 min) administered the cognitive-motor dual-task training. CG - 5 weeks, seven units per week, 1 day for basketball match (2 h) and six times a week for standard basketball training with group basketball training (3 h) and two standard individual training sessions (30 min) including physical and technical exercises only.	EG - 5 basketball games, 10 h. A total of 30 basketball training, 90 h. A total of 10 standard individual training sessions with cognitive-motor-dual-task training, 5 h. Total 45 sessions, 105 h. CG - five basketball games, 10 h. A total of 30 basketball training, 90 h. A total of 10 standard individual training sessions with physical and technical exercises only, 5 h. Total 45 sessions, 105 h.	EG - basketball match, basketball team training, individual cognitive-motor dual-task training, CG - basketball match, basketball team training, individual motor training.
Reigal et al., 2019	SelA	9 weeks, three units per week, each lasting 90 min.	27 sessions, 40.5 h.	Attention training program - Rejilla v. 1.0 software, performed eight tasks: cancellation of numbers (in and no pairs and disappearing and without disappearing) in color matrix, 10 × 10 size and 11 × 11 size, and distracting line

EG, experimental group; CG, control group; ExG, explicit learning group; ImG, implicit learning group; AnG, analogy learning group; SelA, selective attention; SusA, sustained attention; RI, response inhibition; WM, working memory; CF, cognitive flexibility; MS, memory span; WS, working speed; PS, processing speed; 3D-MOT, 3D - Multiple Object Tracking task; BATL, Berliner Achtsamkeitstraining zur Leistungsoptimierung, Berlin Mindfulness Based Training for Athletes; CMDT, cognitive-motor dual-tasking training.

Data extraction was performed by the first author using a standardized Microsoft Excel data-charting form. The extracted data were independently checked by a second author for completeness, accuracy, and consistency. Any discrepancies were resolved through discussion until consensus was reached before the final data synthesis.

Step 5: Collating, summarizing and reporting the data. Given the methodological heterogeneity of the included studies, particularly regarding intervention characteristics, study designs, outcome measures, and assessment instruments, a quantitative meta-analysis was not considered appropriate. Therefore, the findings were synthesized using a narrative synthesis.

The extracted data were analyzed descriptively and grouped according to intervention approach, type of attention, intervention duration, intervention frequency, assessment instruments, and reported outcomes. During the synthesis process, interventions with similar theoretical foundations and implementation characteristics were inductively categorized into broader intervention models, including cognitive training, cognitive-motor dual-task training (CMDT), mindfulness-based interventions, and perceptual-cognitive training. This categorisation facilitated comparison across studies despite methodological heterogeneity.

The use of structured summary tables together with narrative synthesis facilitated comparison across studies, identification of methodological patterns, and extraction of intervention characteristics, assessment instruments, and reported outcomes relevant to the research questions.

In accordance with the objectives of a scoping review and the recommendations of the Joanna Briggs Institute and the PRISMA-ScR guidelines, no formal methodological quality assessment or risk-of-bias assessment of the included studies was undertaken. The primary purpose of this review was to map the existing evidence, identify intervention characteristics, summarize assessment instruments and intervention outcomes, and highlight gaps in the current literature rather than evaluate the methodological quality of individual studies.

## 3 Results

As shown in Figure 1, a total of *N* = 878 studies were identified. First, duplicates were excluded. Initially, the EBSCO host database automated inbuilt duplicate selection tool identified *n* = 30 duplicates that were removed. The remaining studies from the EBSCO host database were exported to the Rayyan research tool, similar to the rest of the studies from the remaining five databases. Duplicates were identified using the Rayyan research duplicate selection tool and removed after the study author manually verified that the study was a duplicate. In total, *n* = 217 more duplicates were identified in the research tool. The remaining *n* = 631 studies were screened by their titles in the screening section. In total, *n* = 532 studies were excluded.

Following title and abstract screening, 99 reports were sought for retrieval. Sixteen reports could not be retrieved, leaving 83 full-text reports that were assessed against the predefined eligibility criteria.

At the final stage of the study selection process, an independent researcher was involved to enhance the reliability of study selection. The researcher was given access to the Rayyan database and independently assessed all 83 full-text reports against the predefined inclusion and exclusion criteria. The independent researcher identified 12 reports as potentially eligible for inclusion. The results of the independent assessment were then compared with those of the first reviewer. Six of the 12 reports overlapped with those identified by the first reviewer. The remaining six reports were excluded after discussion because they did not meet the predefined eligibility criteria: four included participants outside the predefined age range and two involved 1-day interventions. Following comparison and discussion, consensus was reached on the final set of six studies included in the review.

After full-text assessment, 77 reports were excluded: 22 because participants did not meet the predefined age criteria and 55 because they did not meet the remaining review eligibility criteria.

Six studies investigated interventions targeting selective and/or sustained attention. Five studies addressed selective attention, while two addressed sustained attention, with one study (Kittler et al., 2022) examining both attentional domains. Two studies also assessed additional cognitive functions: response inhibition, working memory, and cognitive flexibility (Dania et al., 2021), and memory span, working speed, and processing speed (Fleddermann et al., 2019).

Across the six included studies, a variety of assessment instruments and measurement methods were used to evaluate cognitive and sport-related outcomes. These included standardized attention tests, cognitive and visuomotor tasks, sport-specific performance measures, psychological questionnaires, and neurophysiological measures. Selective attention was assessed using several different instruments and tasks, whereas sustained attention was assessed using the d2-R and the Frankfurter Aufmerksamkeits-Inventar 2 (FAIR-2) (see Table 3).

### 3.1 General study descriptions

The characteristics of the studies can be found in Tables 2, 3. An analysis of the data from the pre-selected studies reveals that research on the development of selective and sustained attention in adolescents has been conducted between 2019 and 2023. All studies have been conducted in Europe. Two studies each have been carried out in Greece and Germany, but one in Spain and Italy. A total of 393 adolescents participated in the studies. Across the included studies, participant ages ranged from 11 to 26 years. Five studies included participants within the predefined target age range of 11–20 years. One study Fleddermann et al. (2019) included a broader overall age range (14–26 years); however, the intervention group had a mean age of 16.38 years (SD = 1.70) and was retained because the intervention group was the primary group of interest for the present review. The youngest study participants were 11–12 years old (Lola et al., 2023), while the oldest participants and also with the largest age range were 14–26 years old (Fleddermann et al., 2019).

The largest number of participants (*N* = 137) took part in a study (Kittler et al., 2022) in Germany, involving students from 16 different sports. On the other hand, the smallest number of participants (*N* = 26) took part in a study in Greece (Dania et al., 2021) with students from the University of Physical Education and Sports Science.

One study (Dania et al., 2021) did not specify the gender of study participants, while three studies included all genders (Fleddermann et al., 2019; Kittler et al., 2022; Lucia et al., 2022). In contrast, one study included only girls (Lola et al., 2023) and another only boys (Reigal et al., 2019). When analyzing the data from the five studies where the gender of the participants was specified, *n* = 170 girls and *n* = 197 boys participated in the studies.

Participants represent a variety of team and individual sports. Basketball (*n* = 3), football (*n* = 3), handball (*n* = 1), volleyball (*n* = 3) and one sport each in archery, boxing, fencing, figure skating, gymnastics, ice dancing, hockey, judo, speed skating, swimming and weightlifting.

### 3.2 Description of selective and sustained attention interventions

Among the six studies included in the scoping review, five studies were identified that used interventions to measure the development of selective attention. We divided these interventions into three models: cognitive task model (*n* = 3), cognitive motor-dual task training model (*n* = 1), mindfulness-based training model (*n* = 1). The interventions selected in the cognitive task model were only cognitive tasks that occurred unrelated to the daily training work. The same approach was used in the mindfulness-based training model, where the tasks identified were unrelated to the daily training work. In contrast, the cognitive motor - dual task model combined cognitive tasks with motor tasks.

The cognitive task model included a study by Dania et al. (2021), which looked at a chess intervention for sports students who play basketball, football and handball on a daily basis. All participants involved were beginners in chess. Research suggests that chess training emphasizes selective attention, response inhibition, working memory and cognitive flexibility, which are important for team games (Dania et al., 2021). The 10-week intervention was divided into topics, chess components to be learned, executive functions and specific session dates were set. No specific tasks were set for the control group.

In a study of young volleyball players (Lola et al., 2023), the intervention was based on three different groups: (a) explicit learning group, (b) implicit learning group, (c) analogy learning group. Each of the three groups received their tasks three times a week for 4 weeks. The control group only participates in the study measurements

Another selective attention intervention used computer-based Rejilla 1.0 software with eight tasks. The intervention group performed these tasks three times a week for 9 weeks. Control group continued daily football training (Reigal et al., 2019).

The mindfulness-based model included Kittler et al. (2022). The BATL mindfulness programme was conducted over 6 weeks, with one session per week. The study used a wait-list controlled crossover design, in which the control group subsequently received the mindfulness programme. The intervention group completed six sessions of the BATL mindfulness program, after which the control group completed the program for a further 6 weeks. For the remaining weeks, regular physical activities were carried out, with measurements between them (Kittler et al., 2022).

One study was included in the Cognitive Motor-Dual Task (CMDT) model. Lucia et al. (2022) and her team involved basketball players who used motor training as an intervention. Participants were also involved in weekly games and routine team drills. The experimental group additionally participated in 2 individual sessions of 30 min each, during which they performed Cognitive-motor dual-task training (CMDT). The training was implemented using the Witty-Sem system. The experimental group had to perform six different cognitive-motor tasks, combined with basketball-specific skills - dribbling, agility, accuracy. The control group also participated in individual training, team training and games, but did not complete the CMDT (Lucia et al., 2022).

In the selected studies, only two studies had interventions to develop sustained attention. We also divided these studies into models: a mindfulness-based training model (*n* = 1) and a Perceptual-cognitive training (PCT) model (*n* = 1).

The perceptual-cognitive training model was applied in a study conducted by Fleddermann et al. (2019), who completed an 8-week three-dimensional (3D) multiple object tracking (3D-MOT) training intervention combined with volleyball-specific exercises (block jumps, ball overhead kicks, throwing) and exercises not specific to volleyball. The control group continued with their regular training and did not receive specific interventions or tasks (Fleddermann et al., 2019). In contrast, Kittler et al. (2022) and his team looked at both selective and sustained attention in their study using the BATL mindfulness program (Kittler et al., 2022).

The interventions in all studies were mostly between 4 and 10 weeks.

The Lucia et al. (2022) study has the most sessions: 45 sessions over 5 weeks. They were divided into three different interventions, such as a basketball game, regular basketball trainings with a team and cognitive-motor dual-task training for the experimental group, while individual trainings for the control group. During the 5-week intervention, athletes participated in basketball matches, regular team training, and two individual CMDT sessions per week (Lucia et al., 2022). In contrast, Kittler et al. (2022) reported that the BATL mindfulness programme was conducted over 6 weeks, with one mindfulness session per week. The study only reported the mean number of hours of exercise per week (*M* = 13.10 h, SD = 3.96 h) (Kittler et al., 2022). The other studies generally completed between 6 and 27 sessions.

The duration of the interventions is also different in each study. The shortest time periods of 30 min are given in the Fleddermann et al. (2019), Lola et al. (2023) studies. Lucia et al. (2022) also states in his study that cognitive motor dual-task training lasted 30 min. Dania et al. (2021), on the other hand, indicates that chess training lasted 2 h, while Reigal et al. (2019), in his study, states that the attention training program Rejilla v 1.0. software with eight different tasks was completed in 90 min in each of the sessions. A full description of the selective and sustained attention interventions can be found in Table 4.

### 3.3 Description of reported intervention outcomes

Across the identified studies, improvements in selective and sustained attention were commonly reported following intervention.

Two studies examined sustained attention (Fleddermann et al., 2019; Kittler et al., 2022). Fleddermann et al. (2019) investigated an 8-week perceptual-cognitive training intervention using 3D Multiple Object Tracking (3D-MOT). Sustained attention was assessed using the d2-R test. The intervention group demonstrated improvements in sustained attention following the training, whereas smaller changes were observed in the control group.

Kittler et al. (2022) investigated both selective and sustained attention using the Berlin Mindfulness-Based Training for Athletes (BATL). Attention was assessed using the Frankfurter Aufmerksamkeits-Inventar 2 (FAIR-2). The study reported changes primarily in the quantity of attention, while no comparable changes were initially observed in attention quality. Continued mindfulness practice was associated with further improvements in attentional performance over time.

All six included studies used pre- and post-intervention assessments to evaluate changes in selective and/or sustained attention. Five studies assessed selective attention, while two studies assessed sustained attention, with Kittler et al. (2022) examining both attentional domains. In addition to the pre- and post-intervention assessments, Kittler et al. (2022) included a long-term follow-up measurement to examine changes in attentional performance over time.

Dania et al. (2021) used chess game intervention and tactical game performance in his study. The results showed that the number of errors in the tests decreased significantly after the intervention for the intervention group, while virtually no change was observed for the control group (Dania et al., 2021).

In a study examining different learning methods in young volleyball athletes under normal and stressful conditions, all three experimental groups (implicit, explicit, and analogy learning) demonstrated improvements in accuracy and reaction time following participation in the intervention program. Reported outcomes indicated better performance compared to the control group (Lola et al., 2023).

Lucia et al. (2022) looked at cognitive-motor dual-task training specifically designed for cognitive and physical performance in young basketball players. The results revealed that the experimental group and the control group improved their performance. The experimental group improved their performance in basketball-specific items by 13% more than the control group, while in cognitive performance they improved their performance by 25.8% in accuracy and 5.4% in response speed. However, according to EEG data, the intervention did not affect sensory processing and attentional processing after both tasks but specifically affected decision-making processes in the experimental group.

The team concluded that cognitive-motor training, by stimulating both proactive and reactive cognitive brain functions, may be better than motor training alone (Lucia et al., 2022).

In another study, where young footballers were given a computerized training program to develop selective attention, the experimental group showed better results compared to both the pre-test results and the control group (Reigal et al., 2019).

Overall, two studies used computer-based attention interventions, three studies combined physical and cognitive interventions, and one study used a cognitive intervention only. Despite the diversity of intervention approaches, the included studies generally reported improvements in selective and/or sustained attention. However, the heterogeneity of the studies limits conclusions regarding intervention effectiveness.

### 3.4 Description of the instrument used

The assessment methods used to evaluate cognitive and sport-related outcomes varied considerably across the six included studies. A range of standardized attention tests, cognitive and visuomotor tasks, sport-specific performance measures, psychological questionnaires, and neurophysiological measures were used (see Table 3). In addition to selective and sustained attention, some studies assessed related cognitive functions, including response inhibition, working memory, cognitive flexibility, memory span, working speed, and information processing speed.

Selective attention was assessed using several methods, including the modified Eriksen Flanker Task, the Frankfurter Aufmerksamkeits-Inventar 2 (FAIR-2), volleyball-specific visual-stimulus tasks, a visuomotor discrimination (Go/No-Go) task, and the d2 Attention Test. Sustained attention was assessed using the d2-R and FAIR-2. The considerable heterogeneity of assessment methods across studies makes direct comparison of intervention outcomes difficult and highlights the need for greater standardization of attention assessment in future research.

## 4 Discussion

The aim of this scoping review was to identify and map the available interventions designed to develop selective and sustained attention among adolescent athletes and to summarize their characteristics, assessment methods, and reported outcomes. Specifically, the review sought to identify the range of interventions used to develop these key attentional abilities in this target population while also summarizing the instruments used to measure selective and sustained attention across the included studies. The findings indicate that research in this field remains limited and heterogeneous, with considerable variation in intervention approaches, outcome measures, and sport disciplines. This heterogeneity makes direct comparisons between studies challenging and highlights the current lack of standardized intervention protocols and assessment methods for developing attentional abilities in adolescent athletes.

Our first research question explored what interventions have been used to develop selective and sustained attention among adolescent athletes. The predominance of interventions targeting selective attention suggests that researchers consider this cognitive function particularly relevant for sport performance during adolescence, as athletes are continuously required to rapidly identify, prioritize, and respond to task-relevant information while suppressing irrelevant stimuli in dynamic sporting environments. This interpretation is consistent with previous literature highlighting the importance of selective attention for efficient perceptual and cognitive processing in sport (Silverman, 2010; Wilkes et al., 2022). In contrast, sustained attention has received considerably less attention in the literature, possibly because it is typically investigated under controlled experimental conditions and often requires longer observation periods and more demanding assessment protocols than can easily be implemented in applied sport settings (Wilkes et al., 2022). Consequently, selective attention may be prioritized not only because of its direct relevance to sport performance but also because it can be assessed using widely adopted and well-validated cognitive tasks, such as the Go/No-Go task (Wilkes et al., 2022) and the Stroop test (Takahashi and Grove, 2020).

Across the included studies, cognitive training interventions represented the most frequently investigated approach. This observation is consistent with Posner et al. (2015), who proposed that selective attention can be strengthened through repeated practice of attentional tasks. Such interventions may enhance athletes’ ability to detect, prioritize, and process relevant environmental information while suppressing distracting stimuli, thereby facilitating faster and more accurate decision-making during sport performance (Müller and Krummenacher, 2006; Posner et al., 2015).

A notable finding was that many interventions combined cognitive and physical components, reflecting the growing recognition that cognitive performance is an integral component of athletic success and should therefore be incorporated into regular sport training rather than developed separately (Wu et al., 2024). This trend also indicates an increasing emphasis on designing interventions that better replicate the cognitive and physical demands encountered during real sporting situations.

One of the included studies investigated Cognitive–Motor Dual-Task (CMDT) training, in which cognitive demands were integrated with sport-specific motor training (Lucia et al., 2022). The study reported improvements in both cognitive and basketball-specific performance, suggesting that the integration of cognitive and motor demands may represent a relevant approach for training attention-related processes in adolescent athletes. Recent evidence indicates that CMDT may be particularly relevant in sport because it simultaneously challenges cognitive processing and motor execution under conditions that more closely resemble the demands of competitive performance (Wu et al., 2024). Compared with isolated cognitive or motor training, such an integrated approach may engage executive functions, information processing, attentional control, and decision-making during sport-related movements (Herold et al., 2018; Posner et al., 2015).

The potential relevance of combining physical activity with cognitive demands is also supported by research beyond the studies included in this review. Physical activity has been associated with physiological and neurocognitive mechanisms, including increased cerebral blood flow, arousal, and neuroplastic adaptations, which may support cognitive and attentional functioning (Diamond and Ling, 2016; Donnelly et al., 2016; Voss et al., 2010). Similarly, Benzing et al. (2016) reported improvements in executive functions following a brief exergame-based intervention in adolescents, while Donnelly and Lambourne (2011) described beneficial effects of physically active cognitive training on cognitive performance. However, these findings should be interpreted as complementary evidence rather than direct evidence for the effectiveness of CMDT in adolescent athletes, given the limited number of relevant studies identified in the present review.

Repeated practice under cognitively demanding and sport-specific conditions may also facilitate the gradual transition from conscious attentional control toward more automatic processing, potentially allowing athletes to allocate attentional resources more efficiently during complex sport situations (Magill, 1998; Williams and Davids, 1995; Lola et al., 2021; Enns et al., 2006). From a practical perspective, this may be particularly relevant in open-skill sports such as basketball, where athletes must simultaneously control the ball, monitor teammates and opponents, anticipate changes in play, and continuously adapt their decisions. Such environments require rapid information processing together with the execution of well-learned motor skills (Piasecki et al., 2013). Integrating cognitive demands into sport-specific training may therefore offer greater ecological relevance than cognitive training performed in isolation.

At the same time, simultaneous cognitive and motor demands may initially impair performance because of cognitive–motor interference. Athletes with greater experience and higher levels of motor automaticity may be less susceptible to such performance decrements (Brinkbäumer et al., 2024). Therefore, although CMDT appears to be a promising approach for developing attention-related processes in adolescent athletes, the evidence identified in this review remains limited, and further well-designed intervention studies are needed to determine its effectiveness and optimal training characteristics.

The review also identified mindfulness-based interventions as a complementary approach to attention development. In addition to cognitive exercises, mindfulness programmes typically combine meditation with sport-specific physical activities (Tang and Posner, 2009; Posner et al., 2015). For example, Kittler et al. (2022) incorporated the BATL mindfulness programme into regular sport training and reported improvements in athletes’ ability to discriminate between relevant and irrelevant stimuli. These findings suggest that mindfulness may enhance attentional control by reducing susceptibility to distractions and improving the ability to maintain focus on task-relevant information during performance. One possible mechanism is that mindfulness training strengthens top-down attentional regulation by improving the ability to intentionally direct, sustain, and reorient attention while reducing automatic responses to irrelevant internal and external stimuli (Tang et al., 2015; Malinowski, 2013). In addition, regular mindfulness practice has been associated with functional changes in brain networks involved in attention and executive control, which may contribute to improved attentional performance in athletes (Tang et al., 2015; Gardner and Moore, 2017). Enhanced attentional control may, in turn, support faster and more accurate decision-making under competitive conditions, where athletes must continuously process relevant information while ignoring distractions (Abernethy, 2001).

Only two studies specifically addressed interventions targeting sustained attention. One possible explanation is that sustained attention is more difficult to investigate in ecologically valid sport settings because it typically requires prolonged observation periods and continuous performance monitoring. Consequently, researchers may preferentially focus on selective attention, which can be more readily assessed during sport-specific tasks. One of the identified interventions used the BATL mindfulness programme to target both selective and sustained attention (Kittler et al., 2022). The intervention demonstrated improvements in sustained attention following the intervention, with further gains observed among athletes who continued practicing independently over time. These findings suggest that sustained attention may benefit not only from structured training but also from continued practice beyond the formal intervention period. The observed benefits are consistent with Kabat-Zinn’s (2015) conceptualisation of mindfulness as purposeful, present-moment awareness characterized by acceptance and non-judgment and with evidence indicating that mindfulness enhances attentional regulation in sport by improving athletes’ ability to maintain focus while reducing susceptibility to internal and external distractions (Bühlmayer et al., 2017).

The second intervention targeting sustained attention involved perceptual–cognitive training (PCT) using a three-dimensional multiple-object tracking (3D-MOT) programme combined with volleyball-specific exercises (Fleddermann et al., 2019). Improvements in perceptual–cognitive performance following the intervention suggest that training attentional processes within dynamic, sport-specific contexts may enhance athletes’ ability to process complex visual information efficiently. From a practical perspective, perceptual–cognitive training may improve visual search strategies, anticipation, and rapid decision-making under competitive conditions by repeatedly exposing athletes to dynamic visual environments that resemble real game situations. This ecological approach may explain why perceptual–cognitive interventions are increasingly incorporated into athlete development programmes, particularly through technologies such as virtual reality and three-dimensional multiple-object tracking systems (Romeas et al., 2019).

Overall, the findings indicate that cognitive training, cognitive–motor dual-task training, mindfulness-based interventions, and perceptual–cognitive training represent the principal approaches currently used to develop selective and sustained attention in adolescent athletes. However, the limited number of available studies, particularly those targeting sustained attention, together with the methodological heterogeneity of intervention protocols, indicates that further high-quality research is required before firm conclusions regarding their comparative effectiveness can be drawn.

Secondary research questions were*: What assessment instruments have been used to measure selective and sustained attention* and *What outcomes have been reported following these interventions?* Although no single assessment tool predominated across the included studies, the d2 and d2-R tests were the most frequently used measures of selective and sustained attention. The diversity of assessment methods reflects the absence of a standardized approach for evaluating attentional performance in adolescent athletes and highlights the need for greater methodological consistency in future research. However, the relatively small number of included studies should also be considered when interpreting these findings.

The frequent use of the d2 and d2-R tests may be explained by their ability to assess visual scanning speed, processing speed, and selective attention, all of which are essential cognitive functions in competitive sport (Bates and Lemay, 2004). Efficient visual scanning enables athletes to rapidly detect relevant environmental information, monitor teammates and opponents, and make timely decisions under constantly changing conditions. These perceptual processes are particularly important in open-skill sports, where performance depends on the continuous integration of visual information with motor responses (Clark et al., 2012). Consequently, the repeated use of these instruments across studies suggests that they provide ecologically relevant measures of attentional performance in sport settings.

Another frequently used assessment instrument was the Go/No-Go task (Visuomotor Discrimination Task), which was used to assess both selective and sustained attention. Beyond measuring attentional performance, this paradigm evaluates inhibitory control, response selection, and rapid decision-making, which are recognized components of executive functioning. These cognitive abilities are fundamental for successful sport performance because athletes must continuously decide whether to initiate, inhibit, or modify their actions in response to rapidly changing environmental stimuli. Previous research has demonstrated that athletes with stronger executive functions, including inhibitory control measured by Go/No-Go paradigms, generally perform better in sports requiring rapid adaptation and strategic decision-making (Wilkes et al., 2022).

Taken together, these findings suggest that the assessment of attention in adolescent athletes increasingly extends beyond the measurement of isolated attentional processes and incorporates broader aspects of executive functioning that are directly relevant to sport performance. Nevertheless, the considerable heterogeneity of assessment instruments identified in this review makes comparisons across studies difficult and highlights the need for greater standardization of attention assessment protocols in future intervention research.

## 5 Strengths and limitations

A key strength of this scoping review lies in its structured and systematic mapping of interventions targeting selective and sustained attention in adolescent athletes. The review followed established scoping review methodology and the PRISMA-ScR reporting guidelines, providing a transparent overview of the available evidence. Furthermore, the detailed description of intervention characteristics, attention assessment instruments, and study outcomes offers a comprehensive overview of the current state of research in this emerging field. The use of a structured screening and data-charting process, with extracted data independently checked by a second reviewer, further strengthened the methodological rigor and transparency of the review.

However, several limitations should be acknowledged. First, only seven studies met the eligibility criteria, indicating that research on interventions targeting selective and sustained attention in adolescent athletes remains limited. The included studies also showed substantial heterogeneity regarding sport disciplines, intervention characteristics, intervention duration, outcome measures, and assessment instruments, making direct comparisons between studies difficult and preventing conclusions regarding the relative effectiveness of different intervention approaches.

Second, one included study reported a participant age range of 14–26 years, which slightly exceeded the predefined age criterion of this review. Although the intervention focused on competitive youth athletes and the intervention group had a mean age within adolescence, this should be considered when interpreting the findings.

In addition, the search strategy relied on predefined keywords and six electronic databases. Although comprehensive, it is possible that relevant studies indexed in other databases or using different terminology were not identified. The restriction to English-language, peer-reviewed publications and the exclusion of gray literature may also have introduced publication and language bias.

Another limitation of this review is that only studies with accessible full texts were included. Consequently, potentially relevant studies without accessible full texts may have been excluded, which may have introduced selection bias.

Finally, as this review followed a scoping review methodology, no formal methodological quality assessment or risk-of-bias assessment of the included studies was performed. Consequently, the findings should be interpreted as a mapping of the available evidence rather than as evidence of intervention effectiveness.

## 6 Conclusion

This scoping review identified a limited but growing body of evidence investigating interventions designed to enhance selective and sustained attention in adolescent athletes. The findings indicate that cognitive training, cognitive-motor dual-task (CMDT) interventions, mindfulness-based programmes, and perceptual-cognitive training have been explored as potential approaches for developing attentional abilities within organized sport settings. However, the available evidence remains heterogeneous with respect to intervention characteristics, sport disciplines, study designs, and assessment methods, limiting direct comparisons between studies and preventing firm conclusions regarding the effectiveness or relative effectiveness of individual interventions.

From a practical perspective, the findings indicate potential value in integrating cognitive demands with sport-specific physical activities as an ecologically relevant approach to attention training in adolescent athletes. Cognitive, cognitive-motor, mindfulness-based, and perceptual-cognitive exercises may therefore warrant consideration within sport training contexts; however, their effectiveness cannot be established on the basis of the current evidence. Any practical application should consequently be interpreted cautiously given the small number of studies and their methodological heterogeneity.

Future research should prioritize well-designed randomized controlled trials involving larger samples of adolescent athletes, longer follow-up periods, and more standardized intervention protocols and attention assessment methods. Greater attention should also be given to sustained attention, which remains substantially underrepresented compared with selective attention in the current literature.

Overall, this review provides a structured mapping of the currently available evidence on interventions targeting selective and sustained attention in adolescent athletes. By identifying existing intervention approaches, assessment methods, and important gaps in the literature, it provides a foundation for future research aimed at developing and evaluating evidence-informed attention training approaches in sport psychology and youth sport.

## Data Availability

The original contributions presented in this study are included in this article/supplementary material, further inquiries can be directed to the corresponding author.
